# An Empirical Analysis of Income Elasticity of Out-of-Pocket Healthcare Expenditure in Mauritius

**DOI:** 10.3390/healthcare10010101

**Published:** 2022-01-05

**Authors:** Jamiil Jeetoo, Vishal Chandr Jaunky

**Affiliations:** 1 Department of Economics and Statistics, Open University of Mauritius, Reduit 80837, Mauritius; jamiil.jeetoo@gmail.com; 2 Department of Business Administration, Technology and Social Sciences, Luleå University of Technology, SE-971 87 Lulea, Sweden

**Keywords:** income elasticity, non-monotonicity, out-of-pocket healthcare expenditure, pseudo-panel, JEL Code, C23, I10

## Abstract

A free universal healthcare provision exists in Mauritius. Yet the share of out-of-pocket healthcare expenditure out of total household expenditure has been growing over time. This study estimates income elasticity of out-of-pocket healthcare expenditure using Mauritian household data within an Engel curve framework. In the absence of longitudinal data on out-of-pocket healthcare expenditure patterns, the study proposes the application of the pseudo-panel approach using cross-sectional Household Budget Survey waves from 1996/97 to 2017. Income elasticity of out-of-pocket healthcare expenditure is estimated to be 0.938, which is just below unity. This implies that out-of-pocket healthcare demand is not considered to be a luxury, but a necessity in Mauritius. In order to see the differences in income elasticities by income groups, separate regressions are estimated for each income quartile over different years. The results indicate that income elasticities of out-of-pocket healthcare expenditure vary non-monotonically.

## 1. Introduction

Health economics is an increasingly important area of research among academics and policymakers, receiving even more attention with the outset of the COVID-19 pandemic. This branch of economics is a multifaceted subject covering concepts such as efficiency, effectiveness, value and behaviour in the production and consumption of health and healthcare [1,2,3,4,5]. There is now a gradual transition of focus from the analysis of healthcare efficiency towards equity in healthcare provisions, with growing interest to align healthcare costs to household income levels [6,7]. At the same time, there is a shift towards the demand side determinants of healthcare at the household level [7,8,9].

The national healthcare system in Mauritius operates on a dual-track basis encompassing the public and the private sectors. Mauritius has one among the most expensive healthcare systems in Africa. Around 73% of the healthcare needs of the population are managed, free of any user cost, at the point of use, in the public sector, financed by the Beveridge system [10,11]. Under this model, the government raises revenue through taxes and other means to finance the delivery of social services, including health. The remaining 27% of healthcare needs are catered by the private sector on a fee basis, either through out-of-pocket payments, including deductibles, or payments effected by private health insurers [11].

In the public healthcare sector, in 2016, the primary healthcare network comprised 18 area health centres, 116 community health centres, 5 medi-clinics and 2 community hospitals. In 2016, 4,732,358 attendances were recorded at the primary healthcare institutions. In the private sector, there were 17 hospitals in 2016, which catered for some 233,966 patients. The private health sector also comprised 30 private medical laboratories, 3 imaging and diagnostic centres and around 342 pharmaceutical retail outlets in 2016 [11,12].

Mauritius spent an estimated total amount of $600 per capita on healthcare in 2017, which is fourfold the amount it was in 2002, where it was estimated to be $136. Out of the estimated amount of $600 per capita, general government healthcare expenditure was $257 and per capita spending on healthcare in the private sector was estimated to be $338. The private sector provides healthcare services on a user fee basis, which is mainly collected through direct out-of-pocket payments and, to a lesser extent, through private voluntary health insurance. It is estimated that 87% of private healthcare expenditure accounts for out-of-pocket healthcare expenditure (OOPHE) and the rest is financed by insurance corporations, non-insurance corporations, NGO’s and the World Health Organisation.

**Figure 1:**
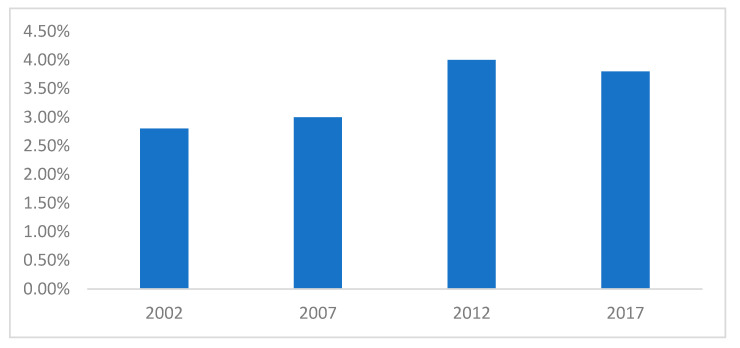
OOPHE out of total household expenditure.

**Figure 2:**
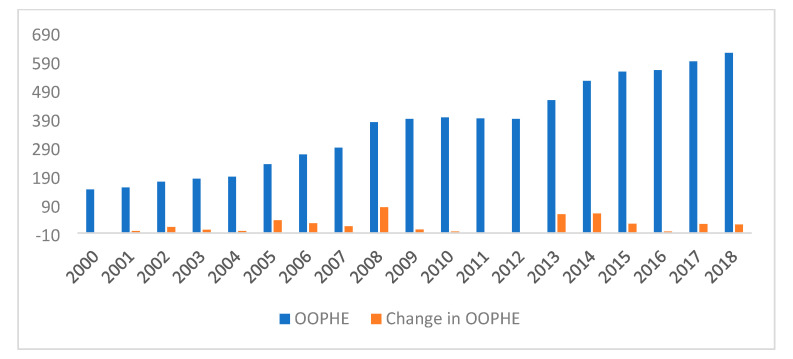
Cumulative change in OOPHE over time.

There has been almost a fivefold increase in OOPHE per capita in Mauritius, from $61 to $293 over the period 2002 to 2017, which corresponds to an increase in the share of OOPHE out of total household healthcare expenditure from 2.8% to 3.8%, as shown in Figure 1. This indicates a trend of the growing importance of OOPHE in Mauritius in healthcare financing over time, while Figure 2 shows the cumulative change in OOPHE over time.

**Figure 3:**
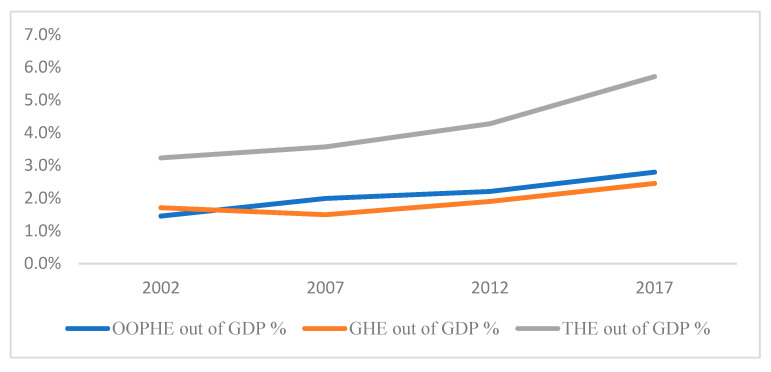
Change in OOPHE, GHE and THE as a percentage of GDP.

**Figure 4:**
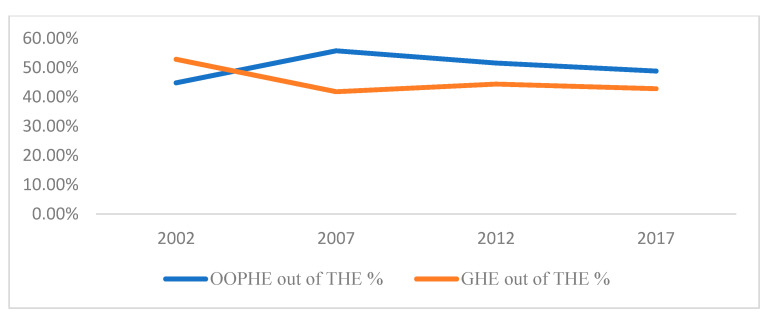
Change in OOPHE and GHE as a percentage of THE.

Figure 3 and Figure 4 show the change in OOPHE as a percentage of GDP and as a percentage of Total Healthcare Expenditure (THE), respectively, over the period 2002 to 2017. It is noted from both figures that after 2002, OOPHE has surpassed Government Healthcare Expenditure (GHE). While the total number of inpatient admissions in government hospitals rose slightly from 186,424 in 2003 to 199,079 in 2018, total number of admissions in private clinics rose by ten-fold from 27,916 in 2003 to 263,376 in 2018. The above trend indicates the shift in the significance of healthcare financing from the public sector to the private sector. If these trends persist, they can erode the willingness to pay of the middle class and the rich to pay taxes for public healthcare services, as they may increasingly opt out of the public healthcare system. This can act as a threat to the sustainability of free public healthcare [9]. From a policy perspective, the government may consider improving nonclinical aspects of public healthcare quality by adopting quality improvement strategies. The results have general implications for emerging countries with a free healthcare policy.

According to the Mauritius National Health Accounts 2017, the increase in OOPHE can be attributed to the increasing per capita income [11]. As per Nundoochan [13], the phenomena of rising OOPHE, which brings in its wake catastrophic health expenditure and impoverishment, could be partially explained by increasing unmet demands of patients attending public facilities as a result of the inefficient use of resources and who in turn have recourse to private healthcare against payment. As explained by Mahumud et al. [8], payment out-of-pocket is not an equitable or efficient financing mechanism. This expansion can mean growing inequalities in access in the free healthcare system.

Against this background, the main objective of this study is to address the puzzle of increasing households’ OOPHE in a context of a free healthcare policy. The aim here is to investigate the income elasticity of healthcare expenditure, which if per se is above unity, would indicate a luxury, whilst if below unity, would indicate a necessity. Generally, estimates of income elasticity can vary according to the methodology applied such as time-series, cross-sectional or panel data techniques [14,15]. Micro-studies in developing countries have often been limited to cross-sectional studies, possibly due to the scarcity of longitudinal data on healthcare expenditure pattern.

This paper aims to present new estimates of the income elasticity of out-of-pocket health expenditure in Mauritius. A pseudo-panel approach is used because of the lack of panel data at the individual level. This allows the identification of cohorts by a set of observed characteristics that are stable over time, and this makes it possible to capture, by a cohort fixed-effect, some unobserved characteristics that could otherwise result in biased estimations. As noted by Di Matteo [16], the relationship between healthcare expenditure and income is not linear and income elasticity of health expenditure is sensitive to the level of analysis, range of income and other economic factors. As such, estimates of the income elasticity of OOPHE may not approximately be linear and constant over time. Given that people in different income levels within a country may demand healthcare differently and they may also respond differently to their income changes, the study uses data from the 1996/97, 2001/02 and 2007, 2013 and 2017 waves of Household Budget Surveys in Mauritius and estimate Tobit regressions of real OOPHE (household healthcare expenditures) by income groups using a number of household characteristics. In particular, it seeks to find out whether the patterns of income elasticity of OOPHE differ by income groups and to what extent and in which direction, if income elasticities of OOPHE have evolved over time.

The remaining of the paper is organised as follows: Section 2 reviews the literature of the income elasticity of healthcare expenditure. Section 3 presents the data and methodology used for the study. Subsequently, the results are discussed in Section 4. Section 5 provides the strength and limitations of this study and Section 6 concludes with a reflection on the results.

## 2. Brief Literature Review

The health economics literature has covered topics dealing with efficiency, effectiveness, value and behavior in the production and consumption of health and healthcare [1,2,3,5]. Studies on efficiency have concentrated on various aspects of the health system. For instance, Chai et al. [5] evaluate the productivity and efficiency of health production pre- and post-reform periods, compare the effects across all the 31 provinces of mainland China and identify potential determinants. Various other studies have explored the question of how health inputs are used to generate the highest possible outcomes both at the micro level [17,18] and the macro level [19,20,21].

Regarding effectiveness, many studies have focused on economic evaluation, and, in particular, cost-effectiveness analysis has become a fundamental part of technology appraisal processes in a number of countries [22,23,24,25]. For example, Dzolkarnaini and Atkins [3] assess the performance and outcome of health systems in managing diabetes in Latin American and Caribbean countries, with a specific focus on the influence of wealth and expenditure on outcome indicators and cost-efficiency. Another strain of literature relates to the value of healthcare [26,27,28,29].

Healthcare demand has received much attention since the study of Grossman [1]. The health production model of Grossman [1] has been very influential in this field of study and has several unique elements that make it notable. Grossman’s model views each individual as both a producer and a consumer of health. Several studies have estimated the determinants of healthcare demand, with a special attention towards the income elasticity of healthcare. Empirical estimations of the income elasticity of healthcare expenditure as illustrated in the literature differ and are usually contradictory, partly due to the employment of diverse datasets, different data aggregation levels and evolvement of econometric techniques over time [9,15,30,31,32,33,34,35].

At the macro level, while some studies have found income elasticity of healthcare expenditure to be above unity [15,36], others found the contrary, whereby the income elasticity of healthcare expenditure is below unity [37,38,39]. Other studies have employed a disaggregating approach by differentiating between private and public healthcare expenditure in estimating income elasticity [32,40,41]. For instance, Khan and Mahumud [41] show that public healthcare expenditure in the South-East Asian Regional countries is a necessity, while private healthcare is a luxury. Other studies have found that the income elasticity of healthcare does vary with the distribution of the healthcare expenditure growth. Chen et al. [42] and Tian et al. [43] show that the income elasticity of healthcare expenditure increases with higher GDP per capita in OECD countries. In addition, Casas et al. [37] show a slight increase in the income elasticity of the healthcare expenditure over the years in the OECD and Eurozone.

At the micro level, many studies have focused on the income elasticity of OOPHE in developing countries and have relied on cross sectional datasets [7,8,9,10]. Results tend to vary among studies. For instance, Mahumud et al. [10] find income elasticity OOPHE to be below unity in Bangladesh, while Pallegedara and Grimm [9] find OOPHE to be above unity in Sri Lanka. A summary of these studies is provided in Table 1.

Most studies have concluded that income has a positive effect on household healthcare expenditure [48,49]. The income elasticities are found to vary for different income groups and over time. For instance, Zare et al. [45] investigate the relationship between income and healthcare expenditure in urban and rural areas in Iran using the spline and quantile regression techniques and find non-uniform effects of income on health expenditures. The spline regression estimates indicate that the income elasticity is lowest for the poorest Iranians. Their quantile regression model assesses the effect of income at different level of medical expenditure, and their finding suggests that households with lower medical expenses are less elastic. Dubey [35] computes the income elasticity of OOPHE of Indian households both across the income groups using the Spline regression model and across the level of health expenditure based on the quintile regression technique using survey data collected in 2014 and 2018. The study finds healthcare to be a necessary good in all cases, with a significant decline in its income elasticity over time. The changes from 2014 to 2018 are found to make income elasticity higher for the lowest income group compared to other income groups for all forms of healthcare expenditures in rural areas and for outpatient and non-medical expenditures in urban areas.

Studies from developed countries have been able to exploit longitudinal survey data in computing the income elasticity of OOPHE. Tsai [46] uses the US Consumer Expenditure Survey longitudinal data from the 1986–1994 and shows that the out-of-pocket total medical costs, medical service expenses and prescription drug expenses income elasticities equal to about 0.89, 1.03 and 0.91, respectively. There are many attempts to capture the quasi-panel effect of healthcare income elasticity in developing countries as well, due to the lack of longitudinal data. Okunade et al. [44] employ the double-hurdle model regression by pooling a total sample of 1994–2000 household data and found household healthcare expenditure to be a necessity. Zhou et al. [50] employ the pooled OLS technique and estimate income elasticity of healthcare for first outpatient visit, out-patient visits among users and first inpatient visit to be 0.098, 0.136 and 0.521 respectively. Zare et al. [45] ran regressions for each year and compared the results over years for Iran. The study find that healthcare is a necessity for all income brackets, and it further points out that the income elasticity is lowest for the poorest individuals. 

Another potential method that has been neglected in the literature is the construction of a pseudo-panel in the absence of longitudinal data. While the pseudo-panel approach has not been employed to estimate income elasticity of OOPHE, it has been utilised in the health economics literature in studies that are not directly related to out-of-pocket expenditure. Propper et al. [51] model the demand for medical insurance in the UK from 1978 to 1996 using the pseudo-panel methodology, and Breyer et al. [52] use the latter to analyse the effect of growing longevity on German healthcare expenditure by applying data from the sickness fund members over the period 1997–2009. 

## 3. Methodology and Data

### 3.1. Theoretical Model

The theoretical model is based on the Engel Curve Framework, which explains the relationship between household expenditure on a specific commodity and household income [53,54]. Engel Curve Framework can be used to estimate income elasticity household expenditure patterns (See Appendix A for the Engel Curve formulation). As a general formulation, income elasticity is inversely related to income [53,54].

**Figure 5:**
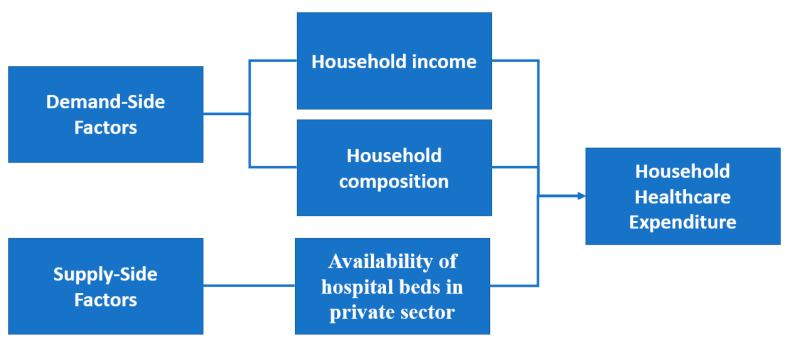
Conceptual Framework.

In addition to income, the study uses a similar conceptual framework to Kumara and Samaratunge [7] by considering other demand-side and supply-side factors. In line with other studies, it includes household composition in addition to household income as a demand-side factor, as well as supply-side factors, represented by the availability of beds in the private sector as determinants of healthcare expenditure [7,9,55]. The conceptual framework is provided in Figure 5. It is hypothesized that household income, household composition and availability affect household healthcare expenditure; this is to be tested in this study.

To analyse the income elasticity of OOPHE in the Engel curve framework, the baseline model is expressed in the double-logarithmic functional form (more details on the general model specification is provided in Appendix B), given that it has proven to be the most appropriate way of estimating elasticities of demand, and it generates more realistic expenditure elasticities [56]. 

### 3.2. Data

The study applies cross-sectional data from the multiple waves of the Household Budget Survey (HBS), carried out by Statistics Mauritius, which are representative samples of the Mauritian population encompassing information on household income, composition and socioeconomic characteristics. The HBS is carried out regularly every five years. Five years of data were chosen from the HBS from 1996/97 to 2017. The previous HBS datasets (prior to 1996) did not include enough details required for the scope of this study. The survey series were carried out on a sample of 6240 (1996/97), 6720 (2001/02), 6720 (2006/07), 6720 (2012) and 7000 (2017) households, respectively. The healthcare expenditure in the HBS refers to mainly out-of-pocket expenditures such as expenditure on therapeutic appliances and equipment, pharmaceutical products, medical services, dental services, paramedical services and hospital services. It excludes private health insurance. Data on the number of hospital beds in the private sector has also been used and was obtained from Statistics Mauritius. The cross-sectional dataset as described above have been used to construct a pseudo-panel which comprises synthetic cohorts as suggested by Deaton [57]. Appendix C provides details on the construction of the Pseudo-Panel used in this study. 

### 3.3. Fixed-Effects Model

The cohort fixed-effects model is applied to the constructed pseudo-panel. The model specification is given in Appendix D. The dependent variable is the mean real OOPHE per capita of cohort c at time t+1 used to capture the effect of income in the previous year on OOPHE per capita. The mean real per capita expenditure of households, the mean household size, the mean crowdedness index and the mean proportion of old people in the household of cohort *c* at time *t*, are included as the independent variables to represent demand side components and total number of hospital beds in the private healthcare sector in Mauritius at time t, are included to represent the supply side factor.

It is to be noted that random effects models are another type of modelling traditionally used on panel data. These models also capture individual effects and are alternative ways of capturing unobserved characteristics of individuals that are fixed over time but have an effect on the variable of interest in the modelling process. However, unlike fixed-effects models, they are based on the assumption that the individual effect is not correlated with the explanatory variables (the individual effect takes into account the correlation of different observations associated with a single individual without overestimating the precision of estimators). If such an assumption cannot be made, there is no point in using pseudo-panels. With independent cross-sections, there is no correlation between the observations, as each individual is only observed once. As such, models can be estimated directly based on stacked individual data [58].

In order to see the differences in income elasticity results by income groups, the study further estimate separate regressions for each income quartiles. The estimations are carried out for periods 1996/97, 2002, 2007, 2012 and 2017 to observe the time dynamics for the income elasticity. Carrying out estimations for different years make it possible to see the evolution of the elasticity through time for different income quartiles. 

### 3.4. Tobit Model

**Figure 6:**
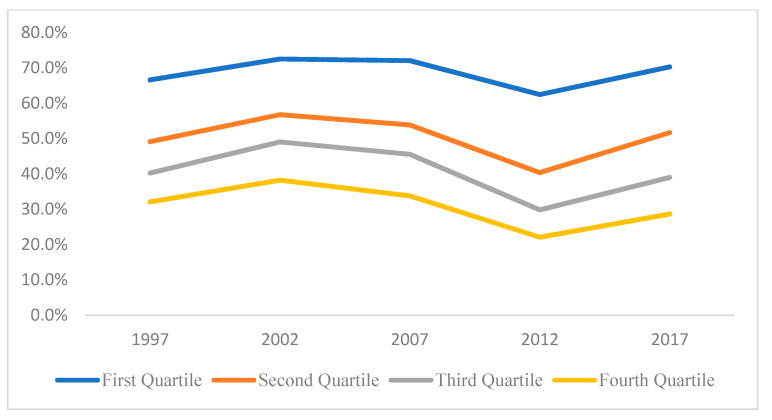
Trend in percentage of households with zero OOPHE by income quartiles.

Because the distribution of OOPHE has a mass at zero, the Tobit analysis is used to estimate separate regressions for each income quartiles using different cross-sectional datasets, which allows for a mass point in the distribution of the dependent variable. Table 2 displays the percentage of households with zero OOPHE by income quartiles while Figure 6 illustrates the trend over time for all the quartiles. The statistical difference between the different income quartiles are tested and are all found to be statistically different; with the t-statistic estimated to be 17.897 (Q1 and Q2), 10.796 (Q2 and Q3) and 12.445 (Q3 and Q4) (all being significant at 1% level of significance).

From Figure 6, it is observed that the percentage of households with zero OOPHE decreases with income. As expected, this shows that poor households (with lower income) are less likely to spend on healthcare compared to richer ones (with higher income). Another important fact is that, for all income quartiles, the percentage of households with zero OOPHE decreases in time until 2012 after which it rose again. This may be interpreted as an increase in number of household spending at least some money on private healthcare from 1996 to 2012 and after that this fell.

The Tobit model also follows the double logarithmic Engel Curve Framework. Appendix E provides the description of the Tobit model [59]. The dependent variable is OOPHE per capita of individual household *i*. The model captures two types of variables: first, the variables regarding household heads (age, gender, education level, marital status and employments status of household head) and second, variables regarding household characteristics (household size, crowdedness index, proportion of old people in the household and region in which the household is located, respectively).

## 4. Empirical Results

Results from the fixed-effects model (Equation (A9)) are given in Table 3. The results indicate that OOPHE has a positive income elasticity, consistent with standard economic theory. The question whether healthcare is a luxury or a necessity has always been of interest, and previous studies found mixed results [9,14,60]. While many studies estimated an income elasticity of healthcare demand in developed countries, there are only a small number of studies that deal with developing countries. In this study, when the pseudo-panel analysis is used, the income elasticity of OOPHE is found to be below but very close to unitary (estimated to be +0.938), which indicates that OOPHE in Mauritius can be classified as a necessity and not a luxury.

Similar to our findings, many studies, using panel data analysis, reveal an inelastic demand for healthcare including Casas et al. [37] in the OECD and Eurozone, Rodríguez et al. [38] for Latin America and the Caribbean countries and for OECD countries Rana et al. [39] for a group of countries. At country level, in the developing world context, other studies find the contrary in Bangladesh [61] and in Nigeria [62]. Using the Bangladesh household income and expenditure survey of 2010 and applying multiple linear regression, Molla et al. [61] find that income elasticities of out-of-pocket healthcare burden are less than unity in Bangladesh; more precisely, it is 0.20. This indicate that there is low flexibility in expenses in relation to income fluctuations, making private healthcare a ‘necessity’. This might be explained by the lack of universal free healthcare coverage in Bangladesh where household OOPHE account for 63.3% total health expenditure, with the second largest financing agent being the government, making up 26.0% of total health expenditure [63]. Likewise, Olasehinde and Olaniyan [62] estimate the income elasticity of household healthcare expenditure to be 0.567 in Nigeria. The study adopts the Engel curve approach and applies the ordinary least squares technique. It is noted that the Nigerian health sector is also dominated by the non-public health system, where private facilities provide almost 80 per cent of health services to Nigerians.

Pallegedara and Grimm [9] identify income as one key driver of rising healthcare expenditures, i.e., as households get richer, they spend an increasing amount on private services suggesting a dissatisfaction with the quality offered by the public sector. Quality improvements in the public sector seem to be necessary to ensure the sustainability of the public health care sector. If the rich and the middle class increasingly opt out of public healthcare, the willingness to pay taxes to finance the free health care policy could shrink [9]. 

Contrary to our results from the pseudo-panel analysis, Rous and Hotchkiss [64] using the Nepal Living Standards Survey, a cross-sectional dataset which is a nationally representative sample of households from 1996, and employing a multi-equation joint estimation model to control for endogeneity of sickness and provider choice, estimate the income elasticity of household health care expenditures to be 1.10. It is noted that unlike Mauritius, at that time Nepal did not have a free healthcare system. It has been argued that private care in developing countries is likely to be a luxury good relative to public care and that, as income rises, households switch from less expensive to more expensive health care alternatives.

As stated earlier, from the literature, the income elasticity of healthcare expenditure is found to be mainly positive. However, the elasticities vary widely across developing countries, and private health is found be either be a luxury or necessity. The main finding of this study shows that OOPHE is a necessity in Mauritius, but such result can neither be generalized to all high-income countries, nor can it be attributed to a particular type of healthcare system.

In addition, from a demand side perspective, the household size variable has a highly significant and negative coefficient; a rise in household size is shown to cause a fall in OOPHE per capita in the household over time. This implies that as household size increases, the demand for resources for alternative purposes increases, and the resources of the household are stretched over a large number of people. This indicates that relatively larger households may not be able to afford spending more on private healthcare, as demand for resources for alternative purposes increases. Zare et al. [45] also report negative impacts of household size on healthcare expenditure in rural areas in Iran. Pallegedara and Grimm [9] also find healthcare expenditures per capita increasing with household size in Sri Lanka. 

The results of this study also indicate that household crowdedness leads to lower OOPHE per capita. This may be explained by the fact that more crowded households lead to less resources and, therefore, less expenditure towards private healthcare expenditure and more reliance on the public healthcare system.

**Figure 7:**
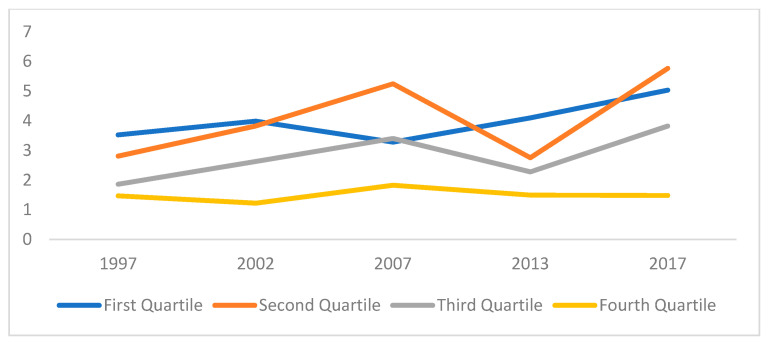
Diagrammatical illustration of the trend of Income Elasticity of OOPHE.

Table 4 provides summary of income elasticity of OOPHE from the Tobit Models corresponding to $\emptyset_{1}$ in Equation (A11). Figure 7 provides a diagrammatical illustration of the trend of income elasticity of OOPHE over time for each quartile and for the overall dataset. Consistent with some previous studies, the study finds that the relationship between income and OOPHE is not constant across all income quartiles [16,45]. 

It is noted that the estimated elasticities are above unitary, which is contrary to the findings of the pseudo-panel analysis. Empirically, the results from the pseudo panel analysis are considered to be more reliable, as opposed to the cross-sectional Tobit models, as it captures the effect of income in the previous year on OOPHE. Nevertheless, the results from the Tobit models are indicatively employed to observe the evolution of income elasticity of each quartile through time. 

Pseudo-panel methods are an alternative to using panel data for estimating fixed-effects models when only independent repeated cross-sectional data are available. They are widely used to estimate price or income elasticities and carry out life-cycle analyses, for which long-term data are required. At the same time, it is noted that panel data have limitations in terms of availability over time and attrition. In these circumstances, the application of pseudo-panel can neutralise specific individual/cohort characteristics [58]. 

From our results in the Tobit models, the magnitudes of elasticity vary widely among the different income quartiles. The results indicate that OOPHE is income elastic at lower levels of income and relatively less elastic at higher levels of income. 

As explained by Di Matteo [16], variation in the magnitude of elasticity is due to the existence of a non-linear relationship between income and health expenditure, and his study on OECD countries suggests that elasticity in low-income groups is higher compared to high-income groups. On the other hand, a long-term study on Iranian households depicts lower elasticity value for low-income groups compared to high-income groups [45]. 

It is notable that studies in the United States, Canada and the OECD countries have also found that the income elasticity of health expenditures is higher for individuals with lower income than people with higher income [16]. Dubey [35] finds income elasticity higher for the lowest income group compared to other income groups for all forms of health expenditure in India. However, Zare et al. [45] find the reverse in Iran, i.e., the income elasticity of medical expenditures is lower for individuals with lower income than people with higher income.

Overall, based on the findings of this study, income elasticity of OOPHE rose over the period 1996/97 to 2017, except that it fell slightly in 2012. This implies that with time, OOPHE increases more rapidly than total expenditure (income).

## 5. Strength and Limitations

As mentioned earlier, the study focuses on the application of the cohort fixed-effect model by constructing a pseudo-panel. The pseudo-panel is constructed using various independent repeated cross-sectional data in the absence of a genuine panel data, which is often the case in developing countries. Pseudo-panel analysis is often used to estimate price or income elasticities and carry out life-cycle analyses, for which long-term data are required, but panel data have limits in terms of availability over time and attrition. It provides an alternative when longitudinal panel data is not available. In the estimations, this makes it possible to capture, by a cohort fixed-effect, some unobserved characteristics that could result in biased estimations. Therefore, the elasticity figure provided from the pseudo-panel model is considered to be more reliable and robust, than when simply using the cross-sectional data analysis [57,58].

While the constructed pseudo-panel suffers less from problems related to measurement error at the individual level, given that they follow cohort means, this also imposes some limitations. First, pseudo-panels do not provide information on intra-cohort mobility [65]. Second, estimates at the cohort level may be a potential source of bias if certain events, such as deaths, affect sizes and composition of cohorts [66]. Third, the construction of a pseudo panel entails a trade-off between the number of cohorts and the number of observations in each cohort. If the number of cohorts is large, estimations will suffer less from small sample problems. Nevertheless, if the size of each cohort is not large enough, average features per cohort will estimate true cohort population averages with a large sampling error [57,65].

## 6. Conclusions

Paradoxically, despite the free healthcare policy, OOPHE is increasing in Mauritius. Strategies to reduce out-of-pocket expenditure are recommended to meet the global commitment to universal health coverage and advocating adjustment in the distribution of available funds [67]. Further understanding of incomes elasticity of OOPHE is essential for creating an effective healthcare system financing policy in Mauritius. Following Costa-Font et al. [60], the value of the income elasticity can provide essential information about the optimum share of coverage of public and private healthcare. If healthcare is a luxury, the concerned healthcare provision may not be required as the private sector will be more efficient in catering for such provisions. The optimal result could be achieved via the invisible hand of market forces. On the other hand, if healthcare is a necessity, then greater state participation and redeployment of healthcare resources are required [33,68].

The purpose of this analysis is to calculate the income elasticities of healthcare expenditure in Mauritius. The contribution of this study is threefold. First, this is the first study of its kind to investigate the income elasticity of OOPHE over time at a household level using the pseudo-panel technique. Second, it is applied to Mauritius, which has some distinctive characteristics with respect to OOPHE and its healthcare system. Third, it analyses the non-monotonicity of income elasticities of OOPHE over different income quartiles and over time. The study is meant to provide policymakers with the understanding of the pattern of the income elasticity elasticities of OOPHE in Mauritius.

The elasticity of out-of-pocket healthcare is found to be below unity, which denotes a necessity and not a luxury good. Its responsiveness being less sensitive to income change can be explained by the fact that Mauritius has been gradually moving towards a high-income country. Nevertheless, policymakers should be cautious in generalizing this study to all developing countries or to countries with free healthcare systems.

The study also allows the elasticities to vary non-monotonically with household income. The findings from the paper suggest that the relationship between health expenditure and income is not a simple linear relationship. Elasticities varies with income levels. The estimated income elasticities fall for successive higher income quartiles. This indicates that households in the lowest income quartiles have higher elasticity than households with higher income. The analysis over time of the relationship of interest suggests that there may be a slight gradual increase in elasticity over time. For all income quartiles the income elasticity of OOPHE rose over time. As such, with the increasing trend in income in Mauritius, it is expected that income elasticity of OOPHE shall continue to rise, where OOPHE will represent a growing share of household expenditure. The marginal increase in OOPHE is anticipated to be higher for households from successive lower income groups, compared to households from higher income groups.

The higher allocation of household budget expenditure to private healthcare services could be explained by the fact that the increase in income over the past 2 decades has allowed people to buy a higher quality of private healthcare services. Given that private healthcare is a necessity good, whereby as income increases, OOPHE rises slower than their income, this also indicates that the quality of healthcare services is important for Mauritians. This could suggest a dissatisfaction with the quality offered by the public sector. If these trends continue, this may erode the willingness to pay of the middle class and the rich to pay taxes for public healthcare services. From a policy perspective, the government may consider improving nonclinical aspects of public healthcare quality by adopting quality improvement strategies. Moreover, the increase in OOPHE could also be partially explained by increasing unmet demands of patients attending public facilities as a result of the inefficient use of resources and who in turn have recourse to healthcare privately against payment. 

In addition, OOPHE is not an equitable or efficient financing mechanism, and the expansion in OOPHE can mean growing inequalities in access to healthcare facilities. As such, strategies to reduce out-of-pocket expenditure are recommended to meet the global commitment to universal health coverage; advocating adjustment in the distribution of available funds [67]. Nevertheless, in setting such strategies, policies should not be limited to nominal healthcare expenditure as countries in Africa are found to be affected by Baumol’s Cost Disease, which indicates that higher expenditure is not necessarily translated into better healthcare services, both in terms of quality and/or quantity [69].

Mauritius was classified as a high-income country in 2019 but was then retrograded to upper-middle income country in 2020, mainly due to the impact of the coronavirus (COVID-19) pandemic. An economic resurgence is expected in the long term, and this could lead a rise in OOPHE. The present study can assist policymakers to make vital decisions when designing OOPHE-relevant regulations. The rising OOPHE is apt to bring about a rise in catastrophic health expenditure and hardships in the wake of the COVID-19 pandemic, unless the quality of public health service is improved. With the prevalence of a Beveridge system, the notion of having a national social health insurance system in parallel has been undermined. The government did announce some years back a voluntary health insurance scheme for public insurance, whereby the former would cover up to 50% of the premium for civil servants [13]. This could help to ease services in the public health sector, as the physician-to-patient ratio could improve.

## Figures and Tables

**Table 1:** Summary Selected Empirical Studies on Income Elasticity of Healthcare Expenditure.

Author	Country	Data	Methodology	Major Findings
Okunade et al. [44]	Thailand	Thailand Socio-Economic Surveys (Period Covered: 1994–2000)	Double-hurdle model	Out-of-pocket healthcare spending behaves as a technical necessity across income quintiles and household sizes.
Zare et al. [45]	Iran	Iran Household Expenditure and Income Surveys (Period Covered: 1984 to 2008)	Spline and quantile regression techniques	Healthcare is a necessity for all income brackets and income elasticity is lowest for the poorest Iranians.
Tsai [46]	US	US Consumer Expenditure Survey (Period Covered: 1986–1994)	Two-stage least squares estimator	Income elasticities of out-of-pocket total medical costs and medical service expenses, and prescription drug expenses are all less than unity.
Kumara and Samaratung [7]	Sri Lanka	Household Income and Expenditure Surveys (Period Covered: 2006–2010)	Probit and Tobit models	The burden of private healthcare is less sensitive towards changes in household income.
Mahumud et al. [8]	Bangladesh	Bangladesh Household Income and Expenditure Survey data (Period Covered: 2010	Ordinary least square method	Income elasticity out-of-pocket healthcare expenditure is below unity.
Pallegedara and Grimm [9]	Sri Lanka	Sri Lanka Household Income and Expenditure Surveys (Period Covered: 1990–2013)	Random-effects regression analysis	Income elasticity for the aggregate of all health care expenditure categories is estimated to be 1.7 (above unity).
Senturk et al. [47]	Turkey	200 households in Turkey	Ordinary least square method	Income elasticity of out-of-pocket healthcare expenditure is estimated to be 0.646 (below unity).
Dubey [35]	India	Indian household survey (Period Covered: 2014–2018)	Spline and quantile regression techniques	Healthcare is a necessity with a significant decline in its income elasticity over time.

Source: Authors’ Own Computation.

**Table 2:** Percentage of households with zero OOPHE by income quartiles.

Percentage of Households with Zero OOPHE by Income Quartiles					
	1996/97	2001/02	2006/07	2012	2017
First Quartile (Q1)	66.6%	72.5%	72.0%	62.4%	70.3%
Second Quartile (Q2)	49.1%	56.8%	53.8%	40.4%	51.7%
Third Quartile (Q3)	40.2%	49.0%	45.5%	29.8%	39.0%
Fourth Quartile (Q4)	32.1%	38.2%	33.8%	22.1%	28.6%

**Table 3:** Fixed-Effects Model Estimates.

Variable	Coefficient
lnRMPCE	0.938 ** (0.444)
lnMHS	−1.530 ** (0.748)
lnMCI	−0.973 *** (0.243)
lnMPO	0.037 (0.092)
lnBED	−1.197 (0.473)
Constant	0.145 (2.927)
R-Squared:	
Number of Observations	67
Within	0.7468
Between	0.9379
Overall	0.8684

Note: Standard errors in parentheses. All independent variables are standardised. **, *** represent *p* < 0.05 and *p* < 0.01, respectively.

**Table 4:** Summary of Income Elasticity of OOPHE from the Tobit Models.

Quartile	1996/97	2001/02	2006/07	2012	2017
First Quartile (Q1)	3.527 ***	3.988 ***	3.282 ***	4.098 ***	5.031 ***
Second Quartile (Q2)	2.810 ***	3.822 ***	5.248 ***	2.754 ***	5.765 ***
Third Quartile (Q3)	1.864 ***	2.630 ***	3.403 ***	2.279 ***	3.822 ***
Fourth Quartile (Q4)	1.473 ***	1.224 ***	1.830 ***	1.496 ***	1.485 ***
Overall	2.236 ***	2.527 ***	2.793 ***	2.511 ***	2.959 ***

Note: *** *p* < 0.01.

## Data Availability

The data presented in this study are available on request from the corresponding author.

## Appendix A

### Engel Curve Formulation

Following Chikobola and Edriss [70], the Engel Curve formulation is derived from the consumer demand theory. Consider a complete system demand equations for *n* goods consisting of the *n* demand equations:

(A1) $$Y_{i}=Y_{j}\;\left(P_{1},\;P_{2},\;\ldots ,\;P_{n},\;I,{µ}_{j}\right),\;j=1,2,3,\ldots ,\;n$$

where $Y_{j}$ is the demand for good *j* by a single household or a group of households, $P_{j}$ is the price of good *j*, *I* is income which is the same as the expenditure on the *n* goods and ${µ}_{j}$ is the stochastic term of the *j*th demand equation. In order to estimate the Equation (A1) it is necessary to specify a particular functional form for the estimation of general relationship. Thus, the *n* demand functions in (A1) are specified as:

(A2) $$Y_{j}=A_{j}P_{1}^{\eta j1}P_{2}^{\eta j2}\ldots .P_{\eta }^{\eta j\eta }I_{\eta }^{\eta j1}e^{µj}$$

The linearization of (A2) by taking logarithms leads to the log-log specification of the form:

(A3) $$lnY_{j\;}=\alpha_{j}+\;\eta_{j1}lnP_{1}+\eta_{j2}lnP_{2}+\ldots +\eta_{jn}lnP_{n}+\eta_{j}ln\;I+{µ}_{j}$$

where $\alpha_{j}=lnA_{j}\;$, considering the price effect constant the $\eta_{j}$ are the income elasticities of demand that can be computed as:

(A4) $$\eta_{j}=\frac{\Delta lnY_{j}}{\Delta lnln\;I\;}=\frac{\Delta Y_{j}}{\Delta I}*\frac{I}{Y_{j}}$$

## Appendix B

### General Model Specification

To analyse the income elasticity of OOPHE in the Engel curve framework, the baseline model is expressed in the double-logarithmic functional form, as below:

(A5) $$lnOOPHE=\Psi \left(lnlNC,\;\Omega \right)$$

where OOPHE represents household healthcare expenditure per capita, *INC* represents household income per capita and Ω is a vector of variables that characterize the control variables. In line with other studies [71,72,73], this study uses household expenditure as a proxy for household income due to the fact that it is regarded in the empirical literature to be a better indicator of permanent income; compared to income, expenditure suffers less from measurement errors. The model is then extended to add the other demand side and supply side variables as provided in the conceptual framework. In line with the conceptual framework (Figure 5), the household composition variables include household size, crowded level and the proportion of old people in households. The supply side variable is the number of hospital beds in the private healthcare sector in Mauritius.

## Appendix C

### Construction of Pseudo-Panel

The first step in the construction of a pseudo-panel is to create homogenous cohorts of respondents depending on similar features being (i) time-invariant and (ii) connected to the dependent variable [74]. For each group there has to be a number of cohorts that is equal to the number of cross-sectional surveys for that particular ‘group’. Older groups would logically participate in more cross-sectional surveys while younger groups would participate in less cross-sectional surveys. Therefore, there are more cohorts for the older groups relative to younger ones. In real panel data, observations of an individual are followed over time. In the pseudo-panel, groups are equivalent to panel units in the pseudo-panel with the cohorts means as observations.

The starting point of panel analysis can be expressed as:

(A6) $$y_{i,t+1}=\beta_{0}+\beta_{1}x_{it}+\mu_{i}+\varepsilon_{it}$$

where i denotes the individuals, t denotes the time period. From the above model, the dependent variable is given by $y_{it+1}$, as it is difficult to observe the effect of the explanatory variables in the same year. The explanatory variables are given by x. $\mu_{i}$ is the unobserved individual specific effect to capture the effect of individual characteristics that are constant over time on the variable of interest, and $\varepsilon_{it}$ is the independent error term, i.e., anything that the model does not take into account. Ignoring the fixed-effect in the estimation leads to biased estimators of the effect of the explanatory variables considered when these variables are correlated with the fixed-effect.

Equation (A6) can be modified to fit the pseudo-panel model and it can be transformed into:

(A7) $${\bar{y}}_{c,t+1}=\delta_{0}+\delta_{1}{\bar{x}}_{ct}+{\bar{\mu }}_{ct}+\varepsilon_{ct}$$

where the subscript i is replaced by c, which represents homogenous groups of individuals. Correspondingly, the variables (including dependent and independent) are converted to mean values for each group and is given as:

(A8) $${\bar{y}}_{c,t+1}=\frac{1}{k}\sum_{i=1}^{k}y_{i,t+1}\;\mathrm{and}\;{\bar{x}}_{ct}=\frac{1}{k}\sum_{i=1}^{k}x_{it}$$

where c denotes the group at time t given and k is the total number of individuals in each cohort. Following Russell and Fraas [75], categorical variables, traditionally used as dummy variables are captured as “proportional variables” in pseudo-panel analysis. These represent the percentage of individuals in a particular cohort with the particular characteristic. ${\bar{\mu }}_{ct}$ represents the average unobserved cohort specific effect varying over time, while unobserved individual effect $\mu_{i}$ does not vary over time. This is because cohorts of each group comprise of distinct members at different points in time. The two main reasons identified by Guillerm [58] for potential source of variation of ${\bar{\mu }}_{ct}$ are (i) the possibility that the cohort is made up of a population itself unstable over time and (ii) sample fluctuation where the individuals that represent it change over time. To eliminate the sources of variation of ${\bar{\mu }}_{ct}$, Guillerm [58] recommends the construction of cohorts (i) which are large enough while conserving variability, and (ii) on a stable population and on the basis of a stable criterion. When a cohort is well constructed, the unobserved cohort effect is considered time-invariant. Therefore, under these conditions ${\bar{\mu }}_{ct}={\bar{\mu }}_{c}$.

According to Deaton [57] cohorts must be adequately large. As a rule of thumb, each cohort should include at least 100 members and there should be sufficient within-group homogeneity and between-group variation [76]. For the sake of this study, birth year, level of education and residential location are the taken as grouping criteria. Education and residential location are assumed to be fixed for each household (only heads as from 24 years were included in the construction of cohort). As noted by Tsai et al. [77], an increase (decrease) in the number of groups leads to a decrease (increase) in cohort size. The number of groups and thresholds within each criterion were chosen in such a way as to ensure consistency over time as well as to optimise the number of observations (of household heads) for all the cohorts included.

Table A1 demonstrates the average cohort size, grouping criteria and the number of cohorts for each group. The age of respondents was collected within different age strings over the repeated survey waves. Birth years were also grouped into year bands. Education (high and low) and residential location (urban and rural) were also used for grouping criteria. This ensures the consistency of the cohorts over time.

**Table A1:** Overview of Constructed Groups Based on Birth Year, Education and Region.

Group	Grouping Criteria			Birth Year	No. of Cohorts
	Birth Year	Education	Region		
1	1940–48	High	Urban	28	5
2	1940–48	Low	Urban	97	5
3	1940–48	High	Rural	67	5
4	1940–48	Low	Rural	172	5
5	1949–57	High	Urban	64	5
6	1949–57	Low	Urban	164	5
7	1949–57	High	Rural	230	5
8	1949–57	Low	Rural	299	5
9	1958–66	High	Urban	113	5
10	1958–66	Low	Urban	256	5
11	1958–66	High	Rural	344	5
12	1958–66	Low	Rural	419	5
13	1967–75	High	Urban	235	5
14	1967–75	Low	Urban	394	5
15	1967–75	High	Rural	488	5
16	1967–75	Low	Rural	475	5
17	1976–84	High	Urban	258	3
18	1976–84	Low	Urban	339	3
19	1976–84	High	Rural	522	3
20	1976–84	Low	Rural	308	3
21	1985–93	High	Urban	178	1
22	1985–93	Low	Urban	171	1
23	1985–93	High	Rural	275	1
24	1985–93	Low	Rural	101	1
Total					96

The constructed pseudo-panel dataset includes twenty-four groups, based on a six by two by two classification of birth year, education and residential location. As long as the group is present in a given survey wave, the cohort is included. Out of the twenty-four groups, sixteen groups include observations over the entire period under study, with each group having five cohorts that correspond to the 1996/97, 2001/02, 2006/07, 2012 and 2017 surveys waves. The more recent the birth years of groups, the fewer observations of cohorts are present. This is possibly due to the heads not yet being born or being below the 24 year-old threshold in earlier survey waves. Ten cohorts that contain fewer than one hundred heads were kept out of the pseudo-panel dataset so as to curtail measurement error. Only 86 cohorts were included in the final dataset.

## Appendix D

### Fixed-Effect Model

The cohort fixed-effects model is applied to the constructed pseudo-panel and is provided as below:

(A9) $$\begin{array}{cc} lnROOPHEPC_{c,t+1}\\ & =\beta_{0}+\beta_{1}lnRMPCE_{ct}\\ & +\beta_{2}lnMHS_{ct}+\beta_{3}lnMCI_{ct}+\beta_{4}lnMPO_{ct}+\;\beta_{5}lnBED_{t}+{\bar{\mu }}_{c}+\varepsilon_{ct} \end{array}$$

For *c* = 1, …, *C* and *t* = 1, … *T*, ${\bar{\mu }}_{c}$ is the unobserved (considered to be time-invariant as explained earlier) cohort effect, and $\varepsilon_{ct}$ captures the error term.

$ROOPHEPC_{ct+1}$ represents mean real OOPHE per capita of cohort c at time t+1. $RMPCE_{ct}$, $MHS_{ct}$, $MCI_{ct}$ and $MPO_{ct}$ represent the mean real per capita expenditure of households, the mean household size, the mean crowdedness index and the mean proportion of old people in the household of cohort c at time t, respectively, representing demand side components. $BED_{t}$ represents the total number of hospital beds in the private healthcare sector in Mauritius at time t, representing the supply side factor.

Note that ROOPHEPC and RMPCE have been deflated using Consumer Price Index data. The income elasticity is given directly by coefficient $\beta_{1}$ in the above model. The crowdedness index is calculated as the number of household members per total living room.

## Appendix E

### Tobit Model

The Tobit model [59] describes the relationship between a non-negative dependent variable $y_{i}$ and an independent variable (or vector) $x_{i}$. As noted by McDonald and Moffit [78], a simple Tobit model can be written as

(A10) $$y_{i}=\left\{\begin{array}{c} y_{i}^{*}\;if\;\;\;y_{i}^{*}>0\\ 0\;\;\;if\;\;y_{i}^{*}\;\leq 0 \end{array}\right.$$

where $y_{i}^{*}$ is a latent variable $y_{i}^{*}=\emptyset x_{i}+\mu_{i}$, assuming $\mu_{i}\sim \;N\left(0,\sigma^{2}\right)$

In this study, the Tobit model also follows the double logarithmic Engel Curve Framework as given below:

(A11) $$\begin{array}{cc} lnOOPHEPC_{i}= & \emptyset_{0}+\emptyset_{1}lnPCE_{i}+\emptyset_{2}lnHS_{i}+\emptyset_{3}lnCI_{i}+\emptyset_{4}lnPO_{i}+\emptyset_{5}lnR_{i}\\ & +\emptyset_{6}lnHAGE_{i}+\emptyset_{7}lnHGEN_{i}+\emptyset_{8}lnHEDU_{i}+\emptyset_{9}lnHMS_{i}\\ & +\emptyset_{10}lnHES_{i}+\;\mu_{i} \end{array}$$

where $OOPHEPC_{i}$ represents OOPHE per capita of individual household *i* and $PCE_{i}$ represents total household expenditure per capita for household *i*. The equation further captures two types of variables; variables for household heads (age, gender, education level, marital status and employments status of household head represented by *HAGE*, *HGEN*, *HEDU*, *HMS* and *HES*, respectively) and variables regarding household characteristics, including *HS*, *CI*, *PO* and *R*, which represent household size, crowdedness index, proportion of old people in the household and region of the household, respectively) of the individual household *i*.
